# Electrochemical Impedance Spectroscopy as a Tool to Monitor Degradation, Fouling and Mechanical Damage in Ion-Selective Electrode Membranes

**DOI:** 10.3390/s26134272

**Published:** 2026-07-05

**Authors:** Martyna Drużyńska, Nikola Lenar, Beata Paczosa-Bator

**Affiliations:** Faculty of Materials Science and Ceramics, AGH University of Krakow, Mickiewicza 30, PL-30059 Krakow, Poland; druzynska@agh.edu.pl

**Keywords:** electrochemical impedance spectroscopy, ion-selective electrodes, membrane degradation, membrane fouling, mechanical damage

## Abstract

**Highlights:**

**What are the main findings?**

- Electrochemical impedance spectroscopy (EIS) successfully distinguished between membrane fouling caused by environmental exposure and physical membrane damage in graphene-based Pb^2+^-selective ion-selective electrodes.
- River water and synthetic urine induced fouling-related changes in membrane morphology, roughness, and wettability, whereas mechanical damage produced a distinct electrochemical signature associated with loss of membrane integrity.

**What are the implications of the main findings?**

- EIS provides a sensitive, non-destructive method for early-stage assessment of ion-selective membrane condition before substantial deterioration of analytical performance occurs.
- The technique can support quality control, lifetime monitoring, and predictive maintenance of all-solid-state ion-selective sensors operating in environmental and biological samples.

**Abstract:**

Electrochemical impedance spectroscopy (EIS) is a powerful, non-destructive tool for evaluating ion-selective electrode (ISE) membrane condition. This work investigated EIS for identifying degradation mechanisms in all-solid-state Pb^2+^-selective electrodes. Graphene-containing PVC membranes deposited on glassy carbon electrodes were exposed to synthetic urine, river water, and seawater (24 h and 1 week) and to mechanical damage (cutting, needle puncture, or both). Degradation was assessed using EIS, potentiometric measurements, contact-angle analysis, profilometry, and SEM. River water and urine exposure decreased hydrophobicity, increased roughness, and produced fouling deposits. Seawater caused only minor morphological and wettability changes, though impedance data showed increased membrane hydration due to high ionic strength. Mechanical damage substantially disrupted membrane integrity, causing pronounced impedance changes, increased potential drift, and reduced analytical performance. Fouling and mechanical damage produced distinct electrochemical signatures: fouling mainly affected surface properties, while mechanical damage altered the membrane–transducer interface, increasing capacitance and reducing resistance. Notably, needle-punctured electrodes retained a near-Nernstian response despite clear impedance changes and reduced long-term stability, showing that EIS detects defects invisible to conventional calibration. These results confirm EIS as a sensitive method for distinguishing fouling from physical damage, useful for early degradation detection and lifetime monitoring of all-solid-state ISEs.

## 1. Introduction

Ion-selective electrodes (ISEs) are among the most widely used electrochemical sensors for the determination of ionic species in environmental, clinical, industrial, and biological samples [1,2,3,4]. Their popularity arises from their simplicity, low cost, broad dynamic range, and suitability for continuous and in situ monitoring. Modern polymer membrane ISEs typically consist of a plasticized polymeric matrix containing an ionophore, ionic sites, and additional membrane components that together govern the selectivity, sensitivity, and transport properties of the sensor [3,4]. Since the introduction of polymeric membrane electrodes, substantial progress has been achieved in improving analytical performance, lowering detection limits, and enhancing long-term operational stability [3,5].

The analytical performance of polymer membrane ISEs is strongly dependent on the physicochemical properties and structural integrity of the sensing membrane. During prolonged operation, membranes may undergo a variety of degradation processes, including leaching of active membrane components, plasticizer loss, adsorption of organic matter, biofouling, and mechanical damage [2,5,6]. These phenomena can alter ion transport pathways, membrane resistance, and interfacial charge-transfer processes, ultimately leading to deterioration of sensor performance manifested by changes in sensitivity, selectivity, response time, and signal stability [6]. Therefore, the development of reliable methods for assessing membrane condition and identifying early signs of degradation remains an important challenge in the field of potentiometric sensing.

Electrochemical impedance spectroscopy (EIS) is a powerful frequency-domain technique that enables detailed characterization of electrochemical systems by analyzing their response to a small sinusoidal perturbation over a broad frequency range [7,8,9,10,11]. Because electrochemical processes occurring within membranes and at interfaces exhibit different characteristic time constants, EIS allows the separation of individual contributions related to bulk membrane transport, interfacial charge transfer, capacitive behavior, and diffusion phenomena. As a non-destructive and highly sensitive technique, EIS has become an important tool for studying ion transport mechanisms in polymeric membranes and evaluating the condition of electrochemical sensors [11,12,13].

In polymer membrane-based ISEs, impedance spectra are commonly interpreted using equivalent electrical circuits composed of resistive and capacitive elements representing different physicochemical processes occurring within the membrane system [9,14]. The high-frequency intercept is generally associated with the uncompensated solution resistance (Rs), whereas the diameter of the principal semicircle observed in Nyquist plots is often related to the bulk membrane resistance (Rm). Deviations from ideal capacitive behavior are frequently described using constant phase elements (CPEs), which account for membrane heterogeneity and distributed interfacial properties. At lower frequencies, diffusion-controlled ion transport may be represented by a Warburg element, manifested as a characteristic linear region in the Nyquist plot. Variations in these parameters provide valuable information regarding changes in membrane composition, transport properties, and structural integrity, making EIS particularly attractive for monitoring membrane degradation and aging processes [11].

The incorporation of carbon nanomaterials into polymeric ion-selective membranes has attracted considerable attention as a strategy for improving membrane conductivity, charge-transfer characteristics, potential stability, and overall sensor performance [15,16,17,18]. Among these materials, graphene and graphene-based derivatives are particularly attractive owing to their high electrical conductivity, large specific surface area, chemical stability, and favorable interfacial properties [18,19,20]. When dispersed directly within the membrane phase, graphene may influence ion transport pathways, membrane resistance, and capacitive behavior, resulting in measurable changes in impedance characteristics. Consequently, EIS provides a particularly suitable approach for investigating how structural modifications, membrane aging, fouling processes, and mechanical defects affect the electrochemical properties of graphene-modified single-piece ion-selective membranes. Monitoring changes in impedance parameters may therefore provide valuable information regarding membrane integrity and the evolution of degradation processes during sensor operation.

To ensure the long-term reliability of sensors operating in complex environments, advanced characterization techniques are increasingly being integrated into sensor evaluation and quality-control procedures. A particularly important contribution was reported by Radu et al., who demonstrated that impedance spectroscopy can be used as a diagnostic tool for evaluating the functionality of polymer membrane-based ion-selective electrodes [11]. The authors showed that different degradation mechanisms produce distinct changes in impedance spectra. Physical damage to the membrane resulted in the appearance of additional semicircles in Nyquist plots together with a reduction in Warburg impedance, indicating the formation of new low-resistance pathways for ion diffusion. In contrast, biofilm formation generated characteristic impedance responses associated with the development of an additional diffusion barrier and alterations in interfacial charge-transfer processes. The study further demonstrated that EIS could detect the loss of active membrane components, including ionophores and ionic sites, highlighting its potential for non-destructive sensor diagnostics and performance monitoring.

Despite these promising results, there are still relatively few studies that clearly link specific membrane degradation processes with their corresponding impedance features. In particular, more work is needed to understand how membrane condition relates to equivalent-circuit parameters and overall electrochemical behavior. This understanding is important for developing methods that can detect early stages of membrane damage before significant loss of sensor performance occurs. This is especially relevant for membranes containing conductive nanomaterials, where changes in structure and ion transport can lead to complex impedance responses that are still not fully understood.

In the present work, electrochemical impedance spectroscopy is employed to investigate the influence of membrane degradation on the electrochemical characteristics of single piece lead-selective electrodes modified with graphene. Impedance spectra obtained for freshly prepared membranes are compared with those recorded after controlled chemical fouling and deliberate mechanical damage. Changes in Nyquist plots and electrical parameters are analyzed to identify characteristic impedance features associated with different degradation mechanisms. The aim of this work is to evaluate the potential of EIS as a non-destructive tool for assessing membrane condition and detecting early signs of degradation.

## 2. Materials and Methods

### 2.1. Electrode Fabrication and Membrane Preparation

Lead-selective ion-selective electrodes (Pb^2+^-ISEs) were prepared based on glassy carbon disc electrodes of 3 mm diameter that served as the conductive substrate. Prior to membrane deposition, the electrode surfaces were mechanically polished using alumina slurries of decreasing particle size, rinsed thoroughly with deionized water, and methanol, and dried under ambient conditions.

The ion-selective membrane cocktail consisted of poly(vinyl chloride) (PVC) (33.1% (*w/w*%)) as the polymer matrix, plasticizers, a lead-selective ionophore, and an appropriate lipophilic ionic additive. All membrane components were dissolved in tetrahydrofuran (THF) to obtain a homogeneous casting solution. Among membrane components following compound were used: potassium tetrakis(p-chlorophenyl) borate (KTpClPB) as lipophilic salt (0.4% *w*/*w*%), 2-nitrophenyl octyl ether (*o*-NPOE) (32.5% (*w*/*w*%)) and bis(1-butylpentyl) adipate (BBPA) (33% (*w*/*w*%)) as plasticizers and lead ionophore IV (1% (*w*/*w*%)).

To simplify electrode fabrication and improve the electrical transduction between the ion-selective membrane and the conductive substrate, graphene was incorporated directly into the membrane matrix. The conductive graphene phase (3.8 wt.%) was dispersed in the membrane cocktail together with the membrane-forming components prior to deposition. As a result, a single-piece all-solid-state ion-selective electrode was obtained, eliminating the need for a separate intermediate solid-contact layer. The presence of graphene within the membrane enhanced charge-transfer properties and facilitated ion-to-electron transduction throughout the sensing layer while maintaining the mechanical integrity of the membrane. The resulting composite membrane was deposited directly onto the glassy carbon substrate in a volume of 40 µL of sensing cocktail and allowed to dry, yielding a compact Pb^2+^-selective electrode architecture suitable for electrochemical impedance spectroscopy and potentiometric measurements.

The prepared electrodes were conditioned in a Pb^2+^ solution of 10^−3^ M concentration for 1 hour prior to electrochemical and potentiometric measurements. Freshly prepared electrodes served as the control group throughout the study.

A total of twelve identically prepared Pb^2+^-selective electrodes were fabricated and subsequently assigned to environmental exposure and mechanical damage experiments.

#### 2.1.1. Experimental Workflow

**Figure 1:**
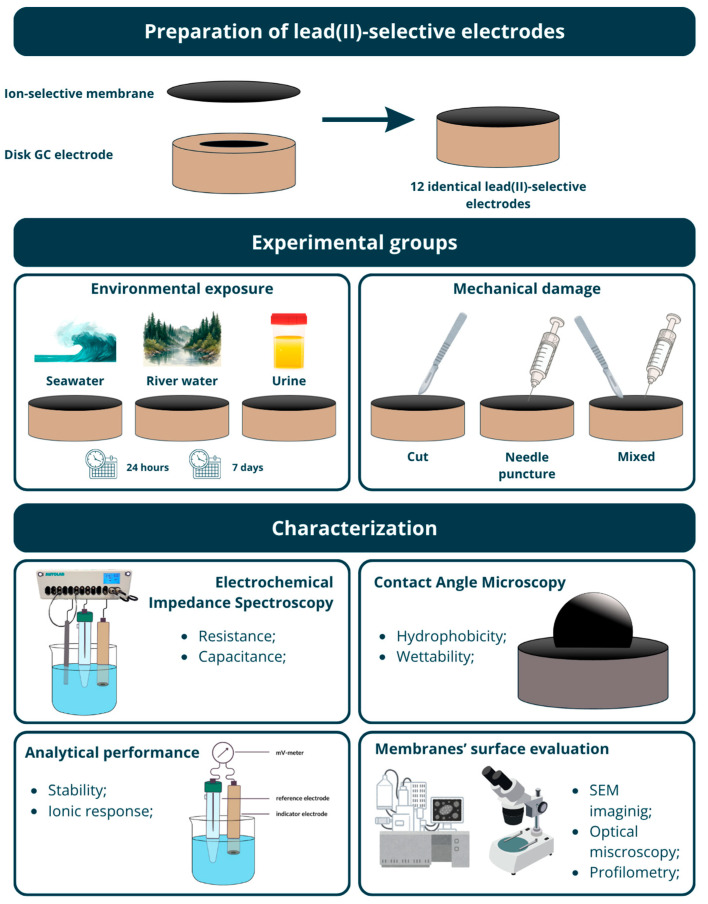
Schematic representation of the experimental workflow for evaluating Pb^2+^-selective electrode degradation.

The comprehensive experimental workflow evaluating the degradation and diagnostic monitoring of the Pb^2+^ -selective electrodes is schematically illustrated in Figure 1.

A total of 12 identical all-solid-state ion-selective electrodes, consisting of glassy carbon disc supports modified with a lead-selective polymeric PVC membrane, were prepared and divided into two primary study groups: chemical fouling and mechanical damage. For the chemical fouling study, nine electrodes were distributed equally (n = 3 per matrix) and exposed to real-world environments: human urine, sea water, and river water, with measurements taken after 24 h and 1 week of exposure. For the mechanical damage study, three electrodes were deliberately subjected to physical destruction: one by sharp cutting, one by needle stabbing, and one via a mixed protocol combining both methods. Following their respective treatments, all experimental electrodes underwent sequential multi-method analysis: comprising electrochemical impedance spectroscopy (EIS), potentiometric response calibration, contact angle microscopy, profilometry, optical microscopy, and scanning electron microscopy (SEM). The resulting electrochemical, wetting, and morphological data were systematically compared against freshly prepared, non-destroyed control electrodes to establish diagnostic criteria for membrane degradation.

#### 2.1.2. Fouling Agents

The selection of these specific environmental matrices was based on their distinct and complex chemical compositions, which are known to interact aggressively with polymeric membranes.

Urine can significantly influence ion selective polymeric membranes (ISMs) by introducing both surface fouling and bulk membrane interference. Organic components in urine, particularly lipophilic molecules, can be extracted into the hydrophobic PVC based membrane phase, resulting in potential drift and loss of selectivity as shown for polymeric ISEs exposed to biological fluids rich in lipids and hydrophobic peptides [21]. Additionally, proteins and other macromolecules naturally present in urine can adsorb onto the membrane surface, forming a conditioning or fouling layer that perturbs ion transport and lowers sensitivity, similar to the protein adsorption effects reported for Pb^2+^ selective membranes [22] and general biofluid induced membrane fouling [23]. Such adsorbed layers can hinder analyte diffusion, alter interfacial ion activities, and generate unstable potentials. Studies on potentiometric multisensor urine analysis also note that urine contains organic species capable of adsorbing onto ISE membranes, reducing sensor reproducibility, and lifetime [24]. Altogether, urine matrix components can induce biofouling like behavior, combining organic adsorption, lipid extraction, and surface film formation, all of which impair potentiometric performance.

As a urine sample, the synthetic model urine was used to investigate the effect of biological matrix exposure on the ion-selective membranes. The synthetic urine solution was purchased from Synthetic Urine e.K. (Eberdingen-Nussdorf, Germany) and used as received without further modification. Electrodes were immersed in the synthetic urine for the designated exposure periods (24 h or 1 week) prior to electrochemical and surface characterization.

Seawater can strongly affect ion selective polymeric membranes through combined biofouling, organic fouling, and inorganic scaling, all of which destabilize potentiometric responses. Marine biofilms form readily on hydrophobic polymer surfaces, where microorganisms attach and secrete extracellular polymeric substances (EPS) rich in proteins and polysaccharides that build conditioning layers and mature biofilms, reducing stability and altering membrane selectivity [25]. EPS and other biochemicals can adsorb onto the membrane surface or penetrate into the organic membrane phase, changing interfacial ion activity and inducing potential drift, as demonstrated for polymeric ISEs operated in marine conditions [21]. Seawater also contains humic substances and natural organic matter, which can adsorb via hydrophobic and electrostatic interactions, contributing to severe flux decline and additional organic fouling layers [26]. High salinity promotes inorganic particle deposition and co fouling, where inorganic particles become trapped within organic surface films, accelerating fouling and reducing sensor reproducibility [27]. Together, these mechanisms create persistent biofilm and organic–inorganic composite layers on polymeric membranes, impairing stability, selectivity, and long term performance in seawater environments.

Certified reference material (CRM) of heavy metal contaminated seawater (ERM-CA403), obtained from the European Commission Joint Research Centre (Geel, Belgium), was employed as a representative biological matrix to assess its effect on ion-selective membranes. Electrodes were immersed in the CRM seawater solution for defined exposure periods (24 h and 1 week) prior to electrochemical and surface characterization.

River water affects polymeric ion selective membranes primarily through natural organic matter (NOM) fouling, microbial deposition, and inorganic–organic interactions characteristic of surface waters. NOM components, including humic substances, polysaccharides, and proteins, readily adsorb onto hydrophobic polymeric surfaces, forming layers that hinder ion transport and generate unstable potentials [28]. In river systems enriched with dissolved organic matter or algal organic matter, high molecular weight biopolymers (e.g., polysaccharides and proteins) act as dominant foulants, blocking pore structures or accumulating on the membrane surface through carboxyl, hydroxyl, and cationic group interactions. River water also contains microbial cells and EPS—including transparent exopolymer particles (TEP), a key EPS-derived fraction, which contribute to early-stage conditioning films and eventual biofilm formation like other natural waters, reducing membrane permeability and disturbing electrochemical stability [29,30]. Humic acids and other NOM fractions can penetrate polymeric membranes, causing potential drifts and reduced sensitivity in potentiometric sensors, as shown for polymeric ISEs exposed to natural waters. Overall, river water induces a biofouling like phenomenon dominated by NOM adsorption, EPS deposition, and combined inorganic–organic fouling, all of which compromise long term sensor performance.

River water sample was collected from the Rudawa River in Kraków, Poland, and used without further treatment for electrode exposure experiments.

### 2.2. Calibration Protocol and Electrochemical Performance Assessment

The potentiometric performance of the Pb^2+^-selective electrodes was evaluated by constructing calibration curves using a series of lead(II) ion standard solutions prepared by serial dilution. Measurements were performed at room temperature in unstirred solutions covering the concentration range from 10^−8^ to 10^−2^ M Pb^2+^. Prior to calibration, the electrodes were conditioned in a 10^−3^ M Pb(NO_3_)_2_ solution.

Potentiometric measurements were performed for freshly prepared electrodes as well as for electrodes subjected to environmental exposure and mechanical damage. The resulting calibration parameters were compared to assess the influence of membrane degradation on the analytical performance of the Pb^2+^-selective electrodes.

### 2.3. Electrochemical Impedance Spectroscopy (EIS)

Electrochemical impedance spectroscopy (EIS) measurements were performed using an Autolab General Purpose Electrochemical System (AUT302N.FRA2-AUTOLAB, Metrohm Autolab, Barendrecht, The Netherlands) with NOVA 2.1. software. The measurements were carried out in a conventional three-electrode electrochemical cell consisting of the investigated Pb^2+^-selective ion-selective electrode as the working electrode, a glassy carbon electrode as the counter electrode, and an Ag/AgCl reference electrode. Impedance spectra were recorded in a 10^−3^ M Pb(NO_3_)_2_ solution. A sinusoidal excitation signal with an amplitude of 100 mV was applied over a frequency range from 100 kHz to 1 mHz. The acquired spectra were presented as Nyquist plots and subsequently analyzed to determine parameters associated with membrane resistance, charge-transfer processes, and capacitive behavior. Measurements were performed for freshly prepared electrodes, electrodes exposed to urine, seawater, and river water for 24 h and one week, as well as electrodes subjected to controlled mechanical damage. The obtained impedance parameters were compared to evaluate the influence of membrane degradation on the electrochemical properties of the Pb^2+^-selective membranes.

### 2.4. Surface Tensiometry

The wettability of the membrane surfaces was evaluated by contact-angle measurements using an Attension Theta optical tensiometer (Biolin Scientific, Espoo, Finland). Measurements were performed using the sessile-drop method at room temperature. A droplet of ultrapure water (5 μL) was deposited onto the membrane surface using an automated microsyringe, and the droplet profile was recorded with a high-resolution camera integrated into the instrument. Contact-angle values were determined by using instrument software by fitting the droplet shape.

Contact-angle measurements were conducted for freshly prepared electrodes, environmentally exposed electrodes, and mechanically damaged electrodes to evaluate changes in surface wettability associated with membrane degradation. The results obtained were correlated with electrochemical impedance spectroscopy, potentiometric characteristics, and scanning electron microscopy observations.

### 2.5. Profilometry

The surface roughness measurements were performed to assess changes in the membrane surface morphology resulting from exposure to environmental samples and physical damage. The tests were performed using a Hommel-Etamic T8000 (JENOPTIK Industrial Metrology Germany GmbH, Villingen-Schwenningen, Germany) contact profilometer with EVOVIS 2.00.1.00 software. The measurements were carried out in linear mode along the X direction, from left to right, at a scanning speed of 0.50 mm/s. Profile evaluation was carried out with a cut-off wavelength λc of 0.08 mm. The roughness parameters Ra and Rq were determined from five measurements per sample. The surface roughness measurements were performed to assess changes in the membrane surface morphology resulting from exposure to environmental samples and physical damage.

### 2.6. Scanning Electron Microscopy (SEM)

The surface morphology, microstructural distribution, and structural integrity of both the intact and degraded Pb^2+^-selective polymeric PVC membranes were evaluated using a Scanning Electron Microscope-Apreo 2 (Thermo Scientific, Waltham, MA, USA). Prior to the microscopic examination, the glassy carbon disc working electrodes assigned to the chemical fouling experimental groups (after 1 week of storage in urine, sea water, and river water matrices) were gently rinsed with deionized water to remove loosely bound environmental salts or biological residue and thoroughly dried at room temperature. The mechanically damaged electrodes (subjected to cutting, needle stabbing, and mixed destruction) were mounted onto appropriate stubs to directly expose and evaluate the macro-defects and structural tears. To prevent surface charging effects and ensure adequate electrical conductivity under the electron beam, all prepared electrode surfaces were sputter-coated with a thin, uniform layer of gold under vacuum. Micrographs of the PVC membrane matrices were recorded inside the electron microscope sample chamber at varying magnifications ranging from 5000× to 10,000× under an optimized accelerating voltage. The obtained microstructural and physical degradation patterns were subsequently used to correlate membrane morphology changes with the performance criteria evaluated via contact angle microscopy, potentiometric response stability, and electrochemical impedance spectroscopy (EIS).

### 2.7. Optical Microscopy Observations

The macroscopic surface condition and wetting behavior of the Pb^2+^-selective membranes were evaluated via optical microscope observations using a SMZ168 Stereo Zoom microscope (Motic, Xiamen, China). Images of the PVC membrane surfaces were captured before and after the chemical fouling treatments (urine, sea water, and river water) and mechanical damage protocols to document clear structural changes, biofilm deposition, and physical defects. These observations directly complemented the contact angle microscopy, profilometry and SEM microstructural findings.

## 3. Results and Discussion

**Figure 2:**
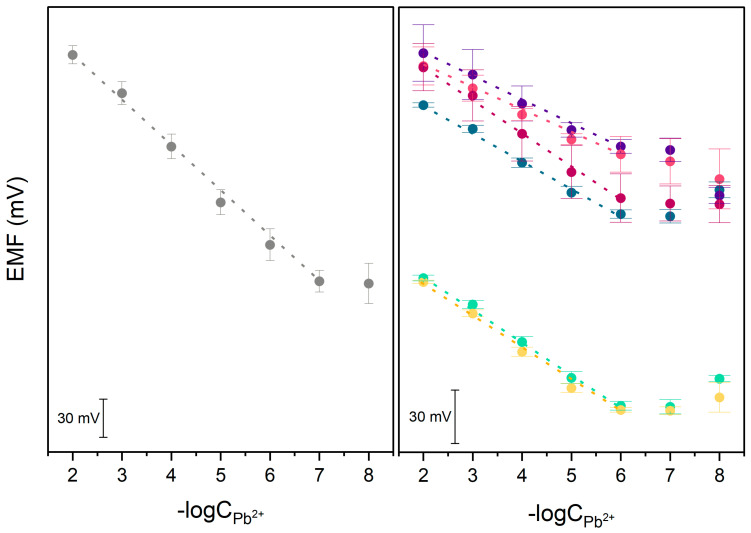
Ionic response towards the Pb^2+^ tested in 10^−2^ to 10^−8^ M standard solutions for control electrode (grey circle), electrodes exposed for seawater (blue circle), river water (green circle), urine (yellow circle) and physically damaged electrodes: punctured (light pink circle), cut (dark pink circle) and mixed (purple circle). Error bars indicate the standard deviation derived from three calibration runs.

In order to evaluate ionic response of designed sensors, the calibrations were conducted. The potentiometric response was registered for optimized Pb^2+^-selective electrodes and the same electrodes exposed to environmental samples and physically destroyed ones. The calibration curves are presented in Figure 2. The calibration parameters of the electrodes, indicative of their analytical performance, are presented in Table 1.

The type of mechanical damage is significant for the analytical utility of electrodes. No significant changes in analytical performance were observed for electrodes with membranes punctured by a needle. In contrast, electrodes with incised membranes showed a marked deterioration in analytical performance, rendering them unsuitable for further use. Notably, needle-punctured electrodes retained a near-Nernstian response despite the physical damage, whereas electrodes with cut membranes failed to produce a reliable potentiometric signal. These findings suggest that puncture-type damage does not compromise the structural integrity of the membrane to a degree that would impair ion transport, while incision causes sufficient disruption to the membrane continuity to preclude analytical use.

Furthermore, the measurement error associated with exposure to environmental samples was considerably lower than that resulting from physical membrane damage, indicating that environmental fouling poses a lesser threat to electrode performance than mechanical destruction. The low deviation of the standard potential observed after exposure to environmental samples indicates high potential stability and confirms the electrode’s resistance to matrix interferences, supporting its suitability for in situ measurements.

**Figure 3:**
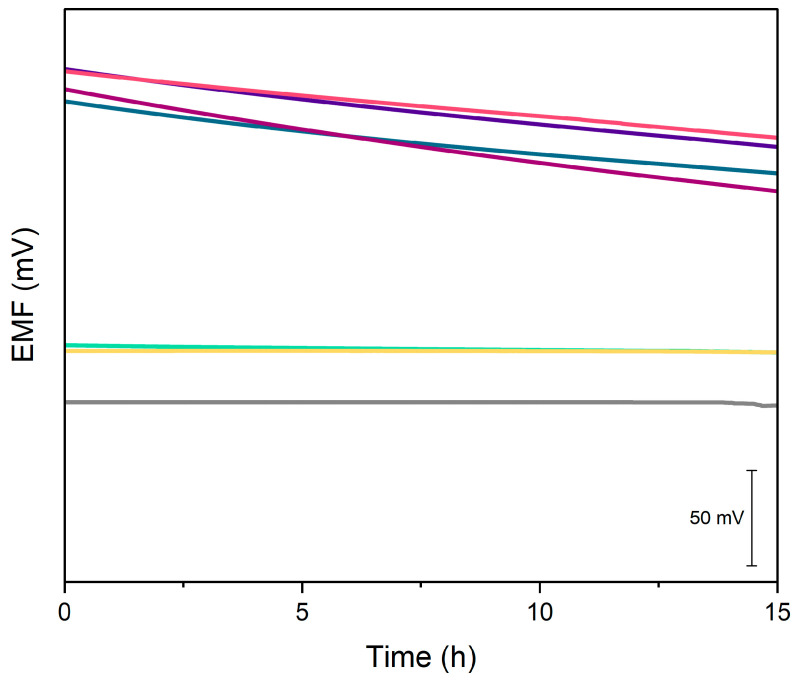
Potentiometric response recorded over a time (15 h) for graphene-based Pb^2+^-ISEs: control electrode (grey line), electrodes exposed for seawater (blue line), river water (green line), urine (yellow line) and physically damaged electrodes: punctured (light pink line), cut (dark pink line) and mixed (purple line).

The potential stability of graphene-based ISEs was evaluated during the long-time potentiometric measurement. The measurement was conducted in 10^−3^ M lead(II) ion solution and lasted 15 h. The average potentiometric signal of optimized lead-sensor was compared with the sensors exposed for environmental samples and physically damaged. The calculated potential drift values were the parameter used to determine the stability of the electrodes. The potential-versus-time dependence is presented in Figure 3. Precise potential drift values, indicating possible instabilities, are shown in Table 2.

The highest potential drift was observed for physically damaged membranes. The obtained values for cut, punctured, and mixed damage were 3.5 mV/h, 2.3 mV/h, and 2.7 mV/h, respectively, confirming the deteriorated analytical properties of the damaged electrodes. Even though needle-punctured electrodes maintained proper analytical parameters, they might not be useful for long-term measurements due to a tendency toward signal instability over time.

Among the electrodes exposed to environmental samples, the highest potential drift was observed for electrodes immersed in seawater for 7 days. This correlates with a slightly lower slope value obtained from calibration curves, compared to electrodes immersed in river water and urine. These results suggest that this electrode might be unfit for long-term monitoring.

The drift values obtained for electrodes immersed in river water and urine are low and comparable to those of the freshly prepared electrode. These results indicate that the designed lead(II)-selective electrodes might be successfully used for long-term monitoring of clinical urine samples and river water.

### 3.1. Evolution of Membrane Resistance and Capacitance

EIS measurements were performed to determine changes in the electrical properties of the electrode. Impedance spectra were recorded for optimized electrodes, electrodes exposed to environmental samples for 24 h, and after one week. In this case, three electrode items were used. For electrodes with physically damaged membranes, spectra were recorded before and after the damage was introduced. Due to poor reproducibility of the damage procedure, measurements were carried out on individual items.

The electrical capacitance values were calculated according to Equation (1), whereas the resistance values were estimated from the diameter of the semicircle in the Nyquist plots.

The electrical capacitance parameter (*C*) was calculated using the following equation:

(1) $$C=\left|\frac{1}{2\pi \cdot f\cdot Z^{\prime \prime }}\right|$$

where *f* stands for frequency and *Z*″ stands for reactance. The resistance parameter values were obtained from the Nyquist plot as a diameter of a semicircle

**Figure 4:**
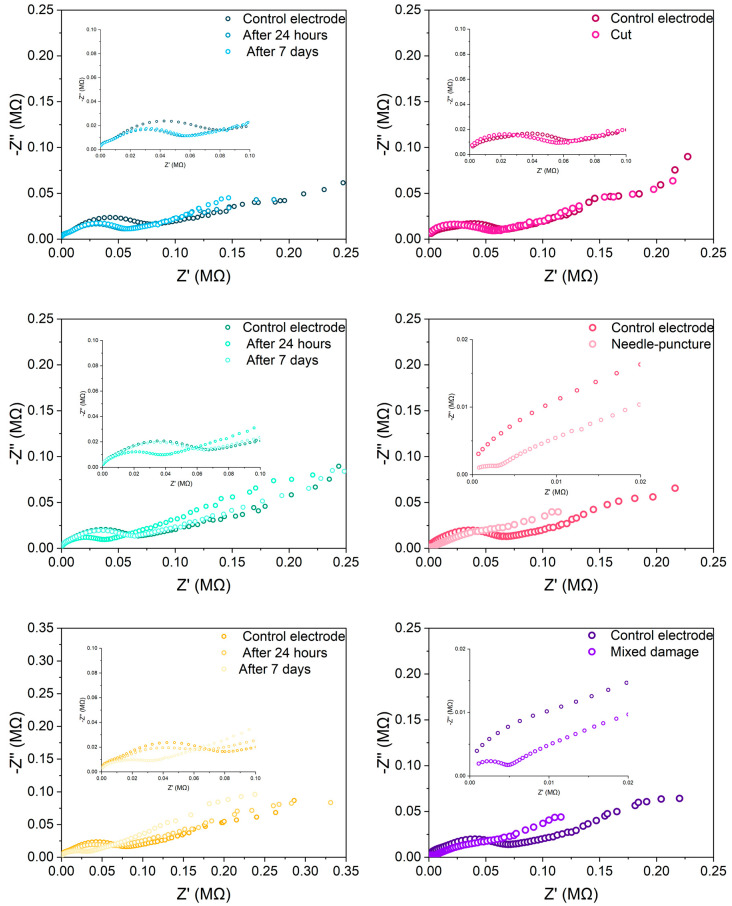
Nyquist plots for ISEs exposed for seawater (blue circle), river water (green circle), urine (yellow circle) and physically damaged electrodes: punctured (light pink circle), cut (dark pink circle) and mixed (purple circle). The insets present a close-up of the high-frequency semicircle region of the Nyquist plot.

Figure 4 shows Nyquist plots, which were used to determine the values of electrical capacitance and resistance. Electrical parameter values obtained via EIS are shown in Table 3.

The EIS results show that the response of the membrane–transducer interface clearly depends on the ionic composition and complexity of the surrounding medium.

The increase in capacitance observed for seawater is consistent with its high ionic strength, which promotes greater hydration and electrolyte uptake into the PVC membrane. As the membrane absorbs more water and ions, its effective thickness decreases and ion pathways within the polymer increase, both of which raise capacitance. This is typical behavior for plasticized PVC membranes under high-ionic-strength conditions and does not necessarily indicate degradation of the graphene layer, since graphene acts only as a passive ion-to-electron transducer. However, as no control experiments employing simple electrolyte solutions of comparable ionic strength were performed, the individual contribution of ionic strength cannot be unequivocally distinguished from other environmental factors. Future studies using model salt solutions without organic constituents will be necessary to verify this hypothesis.

The lack of significant change for river water suggests that its lower ionic strength is not enough to meaningfully affect membrane hydration or ion pathways within the tested timeframe. This indicates good membrane stability under mild environmental conditions.

The decrease in capacitance observed for urine is more likely due to fouling than to hydration effects. Urine contains proteins, urea, and other organic and inorganic compounds that can adsorb onto the membrane surface, partially blocking ion-exchange sites and reducing the area available for charge accumulation. This can lower capacitance even though the membrane remains physically intact. Nevertheless, the relative contributions of ionic composition and surface adsorption phenomena cannot be conclusively separated within the scope of the present work.

The simultaneous increase in capacitance and decrease in resistance for damaged membranes reflects a loss of the membrane’s ion-blocking function at the damaged sites. Physical breaches expose the graphene layer directly to the electrolyte, creating low-resistance, high-capacitance pathways alongside the intact membrane regions. This behaves like a partial short-circuit of the membrane’s barrier properties, with the system increasingly reflecting the graphene/electrolyte interface rather than the bulk membrane.

Overall, these results show that the sensor’s response is governed mainly by fouling under environmental exposure, while mechanical damage produces a more pronounced and distinct electrical signature. This makes EIS a useful tool for distinguishing chemical fouling from physical damage in these sensors.

### 3.2. Surface Wettability

**Figure 5:**
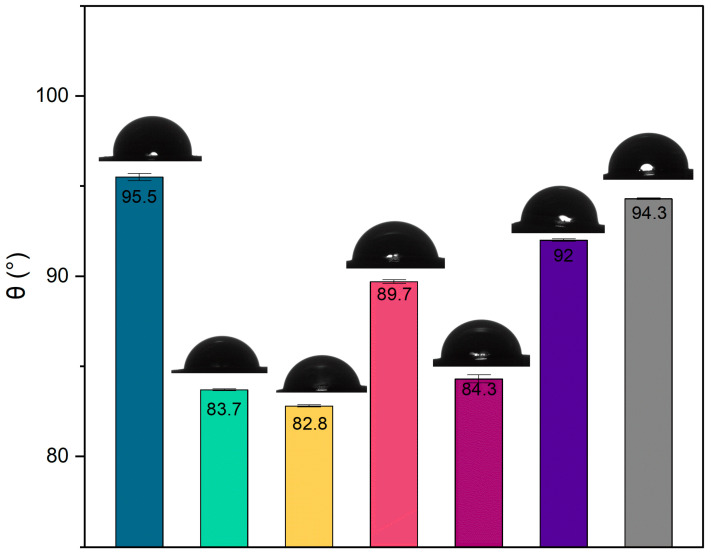
Contact angle values measured for graphene-containing Pb^2+^-selective ion-selective electrode membranes after exposure to different environmental media and mechanical damage: seawater (blue bar), river water (green bar), urine (yellow bar) and physically damaged electrodes: punctured (light pink bar), cut (dark pink bar) and mixed (purple bar). Grey bar represents the contact angle of control Pb^2+^-selective ion-selective electrode.

The contact angle was measured in order to evaluate membrane wettability after exposing it for biological and physical damage. The results are presented in Figure 5.

A water droplet of 5 µL was discharged from a syringe onto the electrode covered with the graphene-based membrane. Hydrophobicity, ensured by graphene nanoparticles incorporated into membrane, of the ion-selective electrode membrane is a key factor contributing to electrode longevity. The wettability test was conducted to assess the influence of biofouling and mechanical damage on membrane condition.

The fresh membrane exhibited a contact angle of 94.3°, confirming its highly hydrophobic character. Exposure to seawater did not significantly affect membrane wettability, yielding a comparable contact angle of 95.5°. In contrast, exposure to river water and synthetic urine resulted in a substantial decrease in contact angle to 83.7° and 82.8°, respectively. These results indicate that prolonged contact with these media increased the affinity of the membrane surface toward water, suggesting changes in surface composition and/or the deposition of fouling layers.

Mechanical damage also affected membrane wettability. The contact angles measured for the cut, punctured, and mixed-damage membranes were 89.7°, 84.3°, and 92.0°, respectively. However, the interpretation of these results should be treated with caution, as the sessile droplet could partially interact with the damaged regions, particularly in the case of cuts and punctures. Such interactions may influence the apparent contact angle and increase measurement variability. Nevertheless, the overall decrease in contact angle relative to the undamaged membrane indicates that mechanical disruption of the membrane structure adversely affects its hydrophobic properties.

From the perspective of potentiometric sensing, maintaining a highly hydrophobic membrane surface is desirable because it limits water-layer formation, reduces fouling susceptibility, and contributes to long-term electrode stability. The observed reduction in contact angle after exposure to river water, synthetic urine, and mechanical damage therefore suggests progressive membrane degradation, which may ultimately affect sensor performance.

### 3.3. Surface Topography

**Figure 6:**
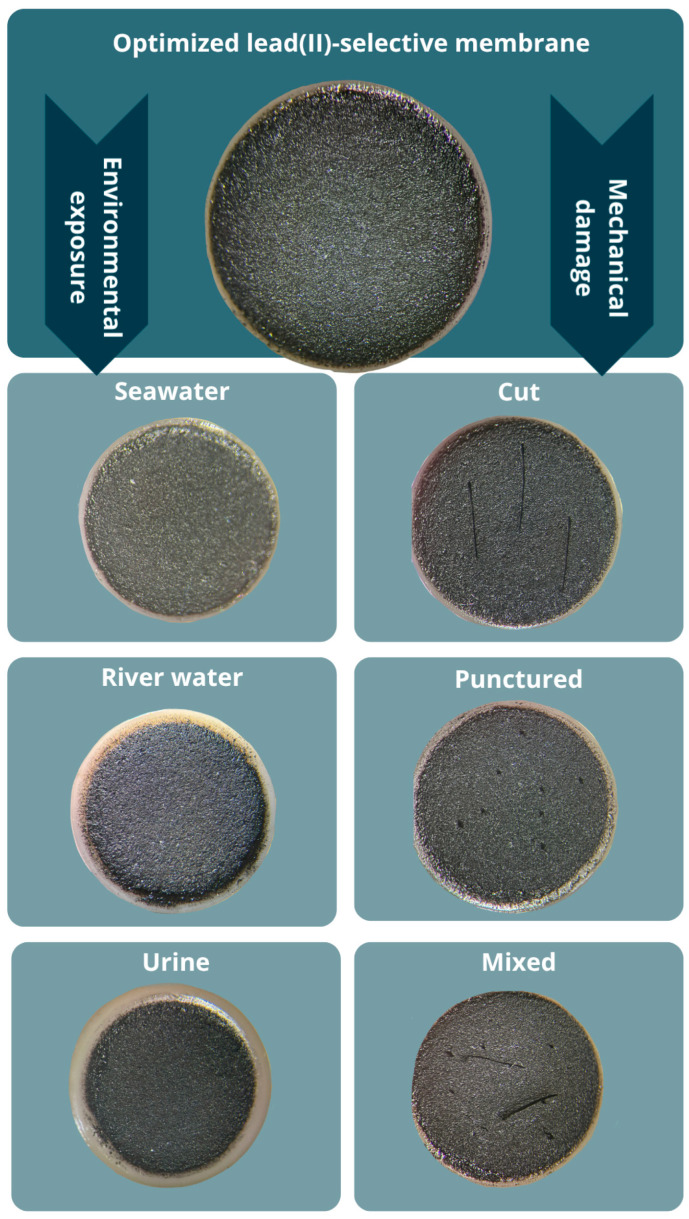
Microscopic images of membranes subjected to environmental sample exposure and mechanical damage (25× magnification).

**Figure 7:**
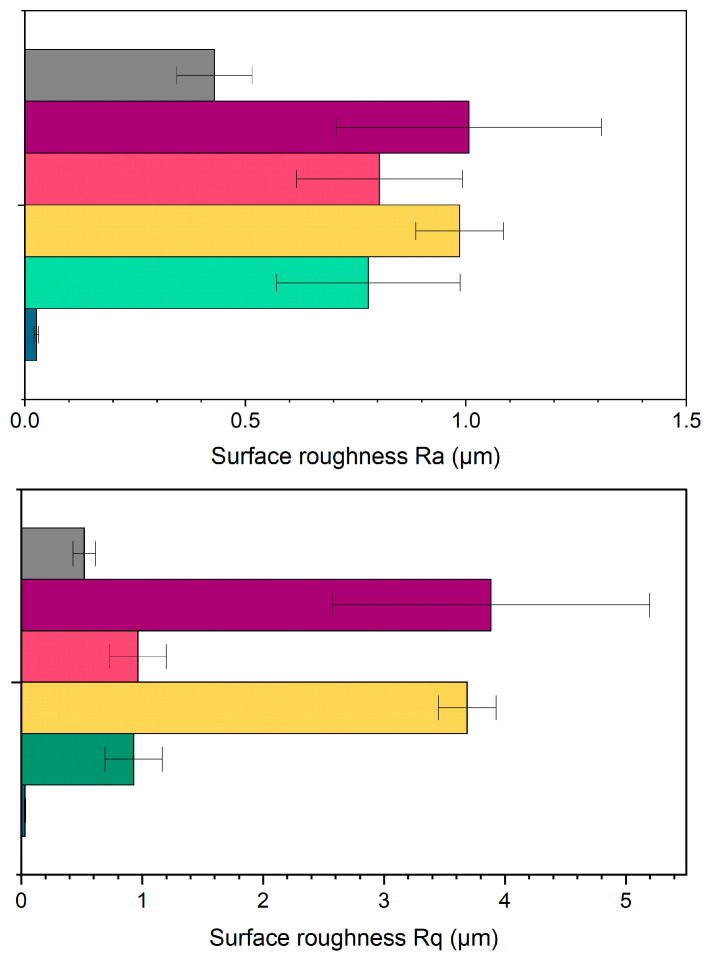
Surface roughness parameters Ra (**top**) and Rq (**bottom**) determined by optical profilometry for graphene-containing Pb^2+^-selective membranes after exposure to: seawater (blue bar), river water (green bar), urine (yellow bar) and physically damaged electrodes: punctured (light pink bar), cut (dark pink bar). Error bars represent the standard deviation of five independent measurements. Grey bar represents the roughness parameters of control Pb^2+^-selective ion-selective electrode.

Surface profilometry measurements were performed to evaluate changes in membrane roughness parameters Ra and Rq. Prior to profilometric analysis, the membrane surfaces were examined using optical microscopy to visualize morphological changes induced by environmental exposure and mechanical damage. Representative optical microscopy images are presented in Figure 6. Subsequently, five profilometric measurements were carried out for each sample. Measurements were performed on optimized membranes, membranes exposed to environmental samples for one week, and physically damaged membranes. The obtained roughness parameters (Ra and Rq) are summarized in Figure 7.

The control membrane exhibited relatively low roughness values, confirming the formation of a homogeneous membrane surface. Exposure to seawater resulted in no significant increase in either Ra or Rq, indicating that the membrane retained its original surface characteristics despite prolonged contact with this environment. This observation is consistent with the contact-angle measurements, which also showed negligible changes in membrane wettability after seawater exposure.

In contrast, membranes exposed to river water and synthetic urine displayed increased roughness parameters. The observed increase in both Ra and Rq suggests partial degradation of the membrane surface, likely associated with fouling processes and interactions between membrane components and constituents present in the environmental samples. Such changes indicate a loss of surface uniformity and may reflect the early stages of membrane deterioration.

The most pronounced changes were observed for mechanically damaged membranes. Both roughness parameters increased substantially following cutting, puncturing, and combined damage, with the mixed-damage membrane exhibiting the highest values. These results confirm the effectiveness of the destruction procedure and demonstrate its significant impact on membrane topography and structural integrity.

From the perspective of potentiometric sensing, increased surface roughness is generally undesirable because it may facilitate water penetration into the membrane structure and promote the formation of an interfacial water layer. Such processes can adversely affect potential stability, selectivity, and long-term electrode performance. The observed increase in Ra and Rq therefore supports the conclusion that both environmental exposure and mechanical damage contribute to membrane degradation.

Interestingly, the roughness measurements correlate well with the wettability results. Membranes exhibiting higher roughness generally showed lower contact-angle values, indicating reduced hydrophobicity. This relationship suggests that changes in surface topography may contribute to enhanced water uptake and altered electrochemical properties of the ion-selective membrane.

Although increased surface roughness is often associated with higher capacitance due to enlargement of the effective surface area, this relationship may not hold for fouled ion-selective membranes. In the present case, the increased Ra and Rq values are attributed to the accumulation of surface deposits rather than the creation of additional electrochemically active sites. Such fouling layers may partially block ion-exchange processes and introduce an additional dielectric barrier, ultimately reducing the capacitance measured by EIS despite the increased geometric roughness.

### 3.4. Morphological Changes

**Figure 8:**
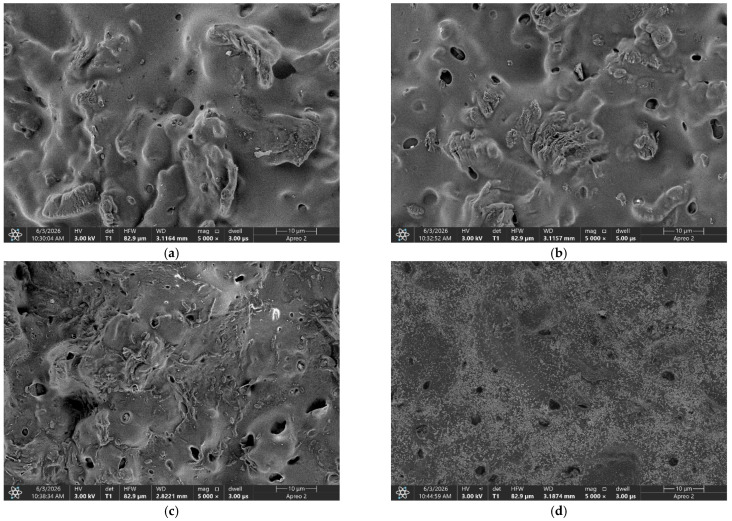

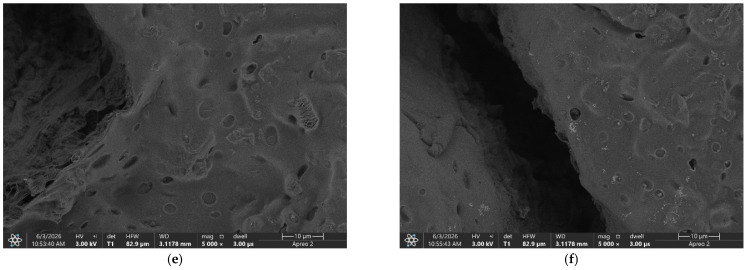
Representative SEM pictures of graphene-containing Pb^2+^-selective membranes: optimized (control) membrane, membranes exposed for one week to seawater, river water, and synthetic urine, and membranes subjected to mechanical damage (puncture and cut). (**a**) control membrane (**b**) membrane exposed to seawater, (**c**) membrane exposed to river water, (**d**) membrane exposed to urine, (**e**) needle-punctured membrane, and (**f**) cut membrane.

Scanning electron microscopy (SEM) was employed to evaluate changes in membrane morphology resulting from environmental exposure and mechanical damage. Representative micrographs of the optimized membrane and membranes subjected to various degradation procedures are presented in Figure 8.

The optimized membrane exhibited a relatively homogeneous surface characterized by a continuous polymer matrix with uniformly distributed graphene flakes embedded within the membrane structure (Figure 8a). The graphene particles appeared well dispersed throughout the PVC matrix, without visible signs of aggregation or phase separation, indicating successful incorporation of the conductive material into the membrane. Only a limited number of pores and surface defects were observed, and the membrane maintained a compact morphology, suggesting good structural integrity prior to exposure. Such a homogeneous distribution of graphene is beneficial for potentiometric sensing, as it promotes efficient ion-to-electron transduction while minimizing uncontrolled water uptake and preserving stable ion-exchange properties.

Following exposure to seawater, only minor morphological changes were observed. The membrane retained a relatively uniform appearance, and no extensive cracking, delamination, or large structural defects were detected (Figure 8b). This observation is consistent with the contact-angle and profilometry results, which indicated that seawater had a limited impact on membrane properties. The SEM images therefore suggest that the membrane remained structurally stable during seawater exposure.

More pronounced morphological changes were observed after exposure to river water. Compared with the optimized membrane, the surface became less homogeneous and exhibited numerous ovals and irregularly shaped deposits distributed across the membrane surface (Figure 8c). These structures were absent in the control membrane and are likely associated with the adsorption of suspended matter and dissolved constituents naturally present in the river-water matrix. Their presence suggests the formation of a fouling layer originating from organic and inorganic components present in the environmental sample. Although the exact nature of these deposits cannot be unequivocally determined based solely on SEM observations, they clearly indicate modification of the membrane surface during exposure. The accumulation of such surface deposits may contribute to the increased roughness and reduced hydrophobicity observed in the profilometric and contact-angle measurements, respectively. From the perspective of potentiometric sensing, the development of a fouling layer is particularly undesirable, as it may alter ion-transport processes at the membrane/solution interface, promote water uptake, and ultimately affect the analytical performance and long-term stability of the electrode.

The membrane exposed to synthetic urine also displayed noticeable morphological alterations as presented in Figure 8d. Compared with the optimized membrane, the surface appeared less homogeneous and exhibited regions covered by irregular deposits and structures of varying contrast. These features were not observed on the fresh membrane and may be associated with the adsorption and accumulation of constituents present in the synthetic urine matrix during prolonged exposure. The presence of these deposits suggests modification of the membrane surface and the development of a fouling layer. The observed features may originate from precipitation or accumulation of inorganic ions and organic compounds present in the model biological fluid. These observations are consistent with the decreased contact angle and increased surface roughness measured for the urine-exposed membrane, indicating deterioration of the original surface properties. Such changes may influence ion transport across the membrane/solution interface and contribute to reduced long-term stability of the potentiometric sensor.

The most severe changes were observed for mechanically damaged membranes. In the punctured (Figure 8e) and cut (Figure 8f) samples, large discontinuities of the membrane structure were clearly visible. The damage introduced defects extending beyond the scale of naturally occurring surface irregularities, resulting in substantial disruption of the membrane continuity. Such defects are expected to facilitate water penetration into the membrane, alter ion-transport pathways, and negatively affect electrode performance. The SEM observations confirm that the mechanical degradation procedure successfully produced significant structural damage to the membrane surface.

Overall, the SEM analysis supports the results obtained by contact-angle measurements and surface profilometry. Samples exhibiting reduced hydrophobicity and increased roughness also displayed greater morphological heterogeneity in the SEM images. The combined results indicate that river water, synthetic urine, and mechanical damage adversely affect membrane integrity, whereas seawater causes comparatively minor changes. These observations further support the use of electrochemical impedance spectroscopy as a sensitive tool for detecting membrane degradation, as structural alterations observed by SEM were accompanied by measurable changes in the electrochemical response of the electrodes.

## 4. Conclusions

The results obtained in this study demonstrate that electrochemical impedance spectroscopy (EIS) is a valuable tool for assessing the condition of graphene-based Pb^2+^-selective ion-selective electrode membranes and for distinguishing between different degradation mechanisms. Changes observed in the impedance response were closely correlated with alterations in membrane morphology, wettability, roughness, and potentiometric performance, confirming that EIS can provide meaningful information regarding membrane integrity and functionality.

Exposure to environmental samples affected the membrane primarily through surface-related processes. River water and synthetic urine induced changes in surface morphology, increased roughness, and reduced hydrophobicity, indicating the development of fouling layers and modification of the membrane surface. Despite these changes, the potentiometric characteristics and long-term stability of the electrodes remained largely preserved, suggesting that the observed degradation was limited to the membrane surface and did not significantly disrupt ion-transport processes within the membrane.

In contrast, mechanical damage produced a markedly different response. Surface profilometry and SEM observations confirmed substantial disruption of membrane integrity, while EIS revealed pronounced changes in the electrical properties of the membrane–transducer interface. The increase in capacitance accompanied by a decrease in resistance indicated the formation of low-resistance pathways through damaged regions of the membrane. These changes were reflected in increased potential drift and deterioration of analytical performance, particularly in electrodes with incised membranes.

Importantly, the type of mechanical damage was found to be critical for sensor performance. Needle puncture did not significantly affect the calibration characteristics of the electrodes, which retained a near-Nernstian response despite visible physical damage. However, EIS measurements and long-term stability tests clearly indicated deterioration of membrane integrity. This finding demonstrates that membrane defects may remain undetected when only conventional potentiometric calibration is employed, whereas EIS is capable of identifying early-stage damage before complete sensor failure occurs.

The combined results further showed that environmental fouling and mechanical damage generate distinct electrochemical signatures. Fouling-related changes were associated mainly with modifications of membrane surface properties, whereas mechanical damage resulted in direct disruption of the membrane barrier and charge-transfer pathways. Consequently, EIS enables differentiation between chemical and physical degradation processes, providing information that cannot be obtained from potentiometric measurements alone.

Overall, the study confirms that electrochemical impedance spectroscopy can serve as a sensitive, non-destructive diagnostic technique for monitoring the condition of ion-selective membranes. The ability of EIS to detect membrane degradation at an early stage, even when the electrode still exhibits acceptable analytical performance, makes this approach particularly attractive for quality control, lifetime assessment, and predictive maintenance of all-solid-state ion-selective sensors.

## Figures and Tables

**Table 1:** Calibration curves parameters.

		Sensitivity S [mV/dec]	Standard Potential ± SD E^o^ [mV] (n = 3)	Linear Range [M]	R^2^
Environmental exposure	Sea	21.81	716.98 ± 2.63	10^−6^–10^−2^	0.9969
	River	25.46	588.89 ± 2.14	10^−6^–10^−2^	0.9973
	Urine	25.5	584.56 ± 2.06	10^−6^–10^−2^	0.9976
Mechanical damage	Mix	17.6	744.15 ± 4.25	10^−6^–10^−2^	0.9755
	Cut	18.83	740.93 ± 2.63	10^−6^–10^−2^	0.9910
	Punctured	26.48	756.7 ± 3.22	10^−6^–10^−2^	0.9961
Control electrode		27.26	792.01 ± 3.55	10^−7^–10^−2^	0.9986

**Table 2:** Potential drifts obtained during long-term stability test.

		Potential Drift [mV/h]
Environmental exposure	Sea	2.5
	River	0.25
	Urine	0.05
Mechanical damage	Mix	2.7
	Cut	3.5
	Punctured	2.3
Control electrode		0.01

**Table 3:** Electrical capacitance parameter values of lead(II)-selective electrodes obtained using an Electrochemical Impedance Spectroscopy.

			Electrical Capacity [µF]	Resistance [kΩ]
Seawater		Control electrode	224	78
		24 h	300	56
		7 days	264	56
River		Control electrode	160	64
		24 h	143	37
		7 days	160	64
Urine		Control electrode	177	70
		24 h	156	64
		7 days	130	32
Mechanical damage	Stab	Control electrode	160	65
		Punctured	222	55
	Cut	Control electrode	216	66
		Cut	302	3
	Mixed	Control electrode	196	69
		Mixed	278	5

## Data Availability

The data presented in this study are available on request from the corresponding author.

## Abbreviations

The following abbreviations are used in this manuscript:

BBPA	Bis(1-butylpentyl) Adipate
C	Capacitance
CRM	Certified Reference Material
EIS	Electrochemical Impedance Spectroscopy
EMF	Electromotive Force
EPS	Extracellular Polymeric Substances
ISE	Ion-Selective Electrode
KTpClPB	Potassium Tetrakis(p-chlorophenyl) Borate
NOM	Natural Organic Matter
*o*-NPOE	2-Nitrophenyl Octyl Ether
PVC	Poly(vinyl chloride)
Ra	Arithmetic Average Roughness
Rq	Root Mean Square Surface Roughness
SEM	Scanning Electron Microscopy
TEP	Transparent Exopolymer Particles
THF	Tetrahydrofuran
